# Baicalein Inhibits Orthotopic Human Non-Small Cell Lung Cancer Xenografts via Src/Id1 Pathway

**DOI:** 10.1155/2019/9806062

**Published:** 2019-03-04

**Authors:** Zhengxiao Zhao, Baojun Liu, Jing Sun, Linwei Lu, Lumei Liu, Jian Qiu, Qiuping Li, Chen Yan, Shan Jiang, Nabijan Mohammadtursun, Wenjuan Ma, Mihui Li, Jingcheng Dong, Weiyi Gong

**Affiliations:** ^1^The Department of Integrative Medicine, Huashan Hospital, Fudan University, 12 Middle Urumqi Road, Shanghai 200040, China; ^2^The Institutes of Integrative Medicine of Fudan University, 12 Middle Urumqi Road, Shanghai 200040, China; ^3^Department of dermatology, Huashan Hospital, Fudan University, Shanghai 200040, China

## Abstract

Non-small cell lung cancer (NSCLC) is one of the most lethal cancers worldwide. Inhibitor of differentiation 1 (Id1) is the member mostly linked to tumorigenesis in Id family and a potential molecular target in cancer therapy. In the current study, we established an orthotopic lung cancer model by injecting athymic nude mice with A549 cells and evaluated the antitumor effect of baicalein and expression of Id1-related proteins* in vivo* and* in vitro*. Micro-CT images showed that tumor volume in baicalein group was significantly reduced. Western blot analysis revealed that baicalein suppressed the expression of Id1 protein, epithelial-to-mesenchymal transition (EMT) related molecules (N-Cadherin, vimentin), and angiogenesis related protein (VEGF-A), accompanied by upregulation of epithelial markers (such as E-cadherin). In addition, phosphorylation of upstream molecular Src was significantly restrained after baicalein treatment. This study firstly demonstrates that baicalein inhibits tumor growth in orthotopic human NSCLC xenografts via targeting Src/Id1 pathway.

## 1. Introduction

Lung cancer is currently the leading cause of cancer-related mortality worldwide, with more than 1.3 million deaths each year [1]. Although many new systemic therapies for treating lung cancer have been developed in recent years, the prognosis has little improvement in the past decades. In particular, lung cancer is notorious for its propensity to develop metastasis. The five-year survival rates for patients with metastatic lung cancer vary globally but are consistently less than 15%, indicating that new approaches are urgently needed for managing lung cancer [2, 3].

Recently, the role of inhibitor of differentiation 1 (Id1) in lung cancer pathogenesis has been gaining interest. Id1 belongs to the helix-loop-helix (HLH) family of transcriptional regulatory proteins which comprises four members, Id1-4, and functions as dominant negative regulators of basic HLH transcriptional factors by preventing them binding to DNA [4]. Among all the Id family members, Id1 is the most linked to carcinogenesis and associated with decreased cell differentiation and induced cell proliferation. Overexpression of Id1 protein has been found in many types of human cancer, which correlated with tumor progression and unfavorable prognosis [5]. Recently Id1 has been shown to be expressed in a variety of tissue microarray samples in non-small cell lung cancer (NSCLC) [6]. Studies have shown that Src signaling pathways are necessary for expression of Id1 [7]. Induction of Id1 has been reported to facilitate the growth and metastasis of NSCLC, while knockdown of Id1 significantly suppresses the proliferation, migration and invasion of NSCLC cells [8, 9]. Genetic loss of Id1 in the host tissue also increased survival and impaired liver colonization of* Id1-/- *lung cancer mice [10]. These studies suggest that therapeutic targeting of Id1 is an attractive strategy for combating lung cancer.

**Figure 1:**
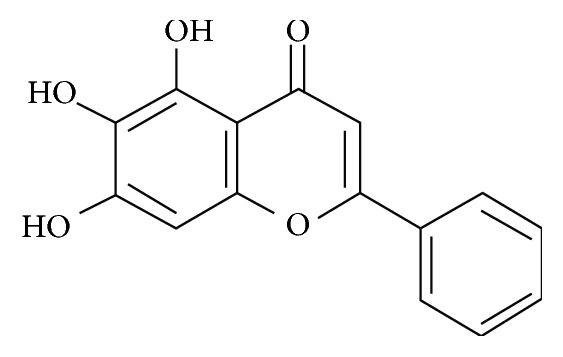
Chemical structure of baicalein.

*Scutellaria baicalensis Georgi *(Lamiaceae family), one of the most popular and multipurpose herbs used in China, has a potential role in the management of cancer [11, 12]. Baicalein (Figure 1) is one of the major bioactive flavones derived from the root of* Scutellaria*. It is cytotoxic to various tumor cells and suppresses tumor growth in vivo without systemic toxicity. Several studies showed that baicalein inhibited lung cancer growth, arrested cell cycle, and decreased metastasis formation [13–16]. Although baicalein has numerous purported properties, the link to Id1 activity in lung cancer is still unknown.

Our published data demonstrated that flavonoid components in* Scutellaria baicalensis* inhibit nicotine-induced proliferation, migration of NSCLC cells, and lung cancer-associated inflammation* in vitro* [14]. In the current study, our purpose was to determine whether baicalein was active against Id1 in orthotopic lung cancer model. Here, we reported, for the first time, baicalein reduced tumor growth of orthotopic human NSCLC xenografts in treated athymic mice. In parallel, baicalein reduced Id1 protein expression, reversed the process of epithelial-to-mesenchymal transition (EMT), and suppressed expression of vascular endothelial growth factor-A (VEGF-A) which is essential to angiogenesis. Furthermore, the above effects of baicalein might be implemented by inhibition of Src phosphorylation. These findings suggest that identification of baicalein as modulators of Id1 function may be a useful strategy in the treatment of cancer.

## 2. Material and Methods

### 2.1. Chemicals

Baicalein (purity > 95%, HPLC) was purchased from Meilun Bio (Dalian, China) and prepared with 0.5% CMC-Na solution. Matrigel was purchased from BD Biocoat (New Jersey, USA). Sodium pentobarbital was from Merck Drugs & Biotechnology (New Jersey, USA). Id1 rabbit monoclonal antibody was from BioChek (San Francisco, USA). Antibodies including anti-E-Cadherin, anti-N-cadherin, anti-vimentin, anti-*β*-actin, anti-Src, and anti-p-Src(Tyr416) were purchased from Cell Signaling Technology (Massachusetts, USA) and VEGF-A from Abcam (Cambridge, UK). Horseradish peroxidase-conjugated goat anti-mouse and anti-rabbit-IgG were from Pierce Biotechnology (Rockford, IL).

### 2.2. Animals

Six-week-old Balb/c male thymic nude mice (weighed 18-20 g) were obtained from B&K Laboratory Animal Co., Ltd. (Shanghai, China). The animals were housed in a temperature-controlled room (22°C) with a 12 h light-dark cycle under pathogen-free conditions and had free access to food and water. All studies were performed in accordance with the recommendations of the Guide for the Care and Use of Laboratory Animals of Fudan University of Chinese Medicine and all procedures were performed under the supervision of the Animal Experimental Ethical Committee of Fudan University (Approval Number: 2015-01-HSYY-DJC-01).

### 2.3. Orthotopic Lung Tumor Model and Treatments

To establish orthotopic xenografts, subconfluent A549 cells were harvested by a brief treatment with trypsin/EDTA, washed with cold PBS by centrifugation, and then resuspended in PBS/matrigel (1:1) and kept on ice before used. The mice were anesthetized with sodium pentobarbital (i.p. 10 mg/kg). A 5 mm incision was made on dorsal side over left lung, about 0.8-1 cm above the lower rib line. Fat and muscles were separated to visualize lung movement. Tumor cells (1 × 10^6^ cells in 50 *μ*L PBS/Matrigel) were injected into left lung parenchyma directly at the depth of 3 mm. The wound was closed with suture, which was removed five days later. Body weights were recorded twice a week.

Mice were randomly assigned to three equal groups (n = 8 per group, 4 per cage): normal, control, and baicalein group. Mice in control group and baicalein group accepted the surgery to establish orthotopic tumor model. Normal group accepted a sham surgery. Thirty days later, baicalein group was intragastrically administered with baicalein (dissolved in 0.5% CMC-Na, 40 mg/kg•d) for 28 days. Control group was intragastrically administered with equal CMC-Na. In addition, 0.9% physiological saline at the same volume was used as a normal control. At the end of the experiment, animals were sacrificed by cervical dislocation and their lungs were harvested and weighted.

### 2.4. Micro-CT Scanning

Micro-CT was performed four weeks later after the start of treatment as described. Briefly, serial lung imaging was scanned on an* in vivo* micro-CT system (SkyScan1076, BrukermicroCT, Kontich, Belgium). Data were acquired at 38 *μ*m isotropic resolution, 49 kV, 200 *μ*A, 360° rotation around the vertical axis, rotation step of 0.5°, and camera exposure time of 250 milliseconds. During* in vivo* imaging, the animals were anesthetized with 2% isoflurane in medical air and kept at a constant 37°C temperature by regulated warm airflow. Acquired projection images were reconstructed with NRecon v.1.6.9 software (BrukermicroCT) with a beam hardening correction of 40% and ring artifact correction of 10, resulting in the acquisition of 518 cross sections per lung.

### 2.5. Immunohistochemistry

Lungs were excised from each mouse, fixed in 4% formalin, and embedded in paraffin for immunohistochemical staining. Briefly, sections 3 um thin cut from blocks were stained with hematoxylin and eosin stain (HE). Immunohistochemical staining was carried out manually using rabbit monoclonal IgGs specific Id1 antibody (1:100; BioCheck). Sections were cut and submerged in EDTA buffer and heated in a microwave oven (100°C) for antigen retrieval. Endogenous peroxidase activity was blocked by 15 min of treatment with 0.3% hydrogen peroxide at 37°C. After rinsing, the sections were further blocked by 30 min of treatment with 5% goat serum at 37°C and then incubated with the primary antibody at 4°C overnight, followed by biotin labeled secondary antibody, developed with 3,3′-diaminobenzidine. For a negative control, a nonspecific antibody was used instead of the primary antigen. Staining was visualized and quantified by light microscope.

### 2.6. Western Blot

Tissues or cells were lysed at 4°C according to instructions of protein extraction kit (Beyotime Biotechnology, Haimen, China). Protein concentrations were determined by BCA protein assay kit (Beyotime). The protein extract (20 *μ*g) was mixed with SDS-PAGE Sample Loading Buffer and heated at 100°C for 15 minutes. Equal amounts of protein were separated by 12% SDS-polyacrylamide gel electrophoresis and transferred to polyvinylidene difluoride (PVDF) membranes. The membranes were then blocked at room temperature for 1 h with 5% (w/v) nonfat milk in TBST buffer and incubated with primary antibodies in 1% BSA overnight at 4°C with continuous shaking. After three washes in TBST, membranes were incubated with secondary antibodies conjugated with horseradish peroxidase for one hand visualized by enhanced chemiluminescence using Supersignal West Femto Chemiluminescent Substrate (Pierce Biotechnology Inc., Rockford, IL, USA). Band intensities were analyzed by NIH ImageJ software and normalized to *β*-actin. The western blot data were replicated three times.

### 2.7. Statistical Analysis

Mean values and standard errors of the mean were calculated for each point from the pooled normalized data. The one-way analysis of variance (ANOVA) test (SPSS software package, SPSS Inc., Chicago, IL, USA) was used for evaluating the differences between groups. Statistical significance (*P*<0.05) was established with* post hoc* comparison by the Dunnett's test, unless otherwise stated. Data were graphed by Prism 6.0 (GraphPad Software, La Jolla, CA).

## 3. Results

### 3.1. Baicalein Inhibited the Growth of Orthotopic Human NSCLC Xenografts

**Figure 2:**
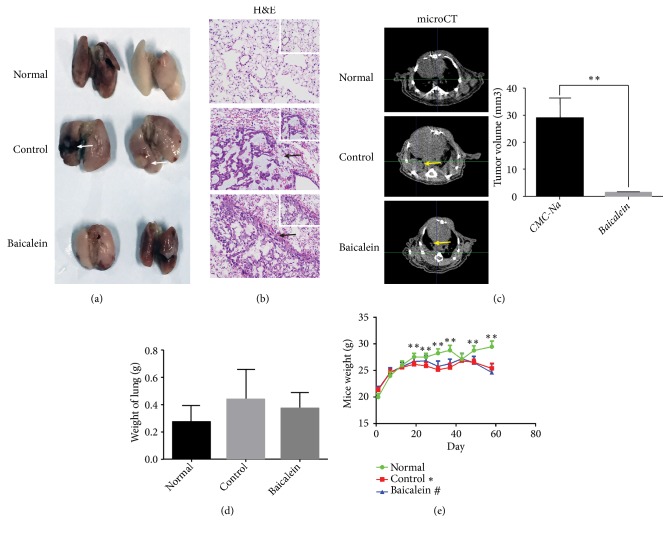
Baicalein inhibited tumor growth of A549 orthotopic NSCLC xenografts. (a) Gross images of normal lungs and tumor bearing lungs. Nodules can be seen in control group (white arrows). (b) Hematoxylin-eosin (H&E) staining (200X, 400X magnifications) of normal lungs and tumor bearing lungs. Black arrows indicated the tumor cells. (c) Representative micro-CT images of mice in each group showed the lung anatomy and tumor load (yellow arrows) after treatment. (d) Weight of lungs. (e) Changes of mice weight in each group over time. Data was shown as mean ± SEM, *∗P*<0.05; *∗∗P*<0.01.

To assess the anti-tumor effect of baicalein, we built an orthotopic lung cancer model in Balb/c nude mice by A549 cells implantation. Except for normal group, mice were injected with 1x10^6^ A549 cells into the left lungs. Thirty days following inoculation, mice in baicalein group were intragastrically administered with baicalein (0.5% CMC-Na solution, 40 mg/kg). Control group was intragastrically administered with CMC-Na (0.5%, 0.2 mL per mouse) and normal group was with 0.9% physiological saline. After 28 days of treatment, mice were scanned with micro-CT and sacrificed. Lungs of mice were harvested; gray nodules could be seen in control group (Figure 2(a), white arrows). In control and baicalein groups, HE staining of lungs showed heterogeneous cells with larger eosinophilic nucleoli and vacuoles in the cytoplasm. Visible glandular cavities could be seen among these cells (Figure 2(b), black arrows) which indicated the success of orthotopic lung cancer model. Micro-CT scanning exhibited much smaller nodules in left lung of baicalein group comparing to control group (Figure 2(c), yellow arrows) and the differences were significant (*P*<0.01). We also measured the weight of lungs in each group. Results showed that the lungs in control group were heavier than the other two groups, but significant difference was not observed (Figure 2(d)). Mice weight in normal group increased gradually over time and was higher than that of control and baicalein-treated groups after 20th day of inoculation; the difference between normal group and control group was significant (*P*<0.01) (Figure 2(e)).

### 3.2. Baicalein Inhibited Id1 Expression

**Figure 3:**
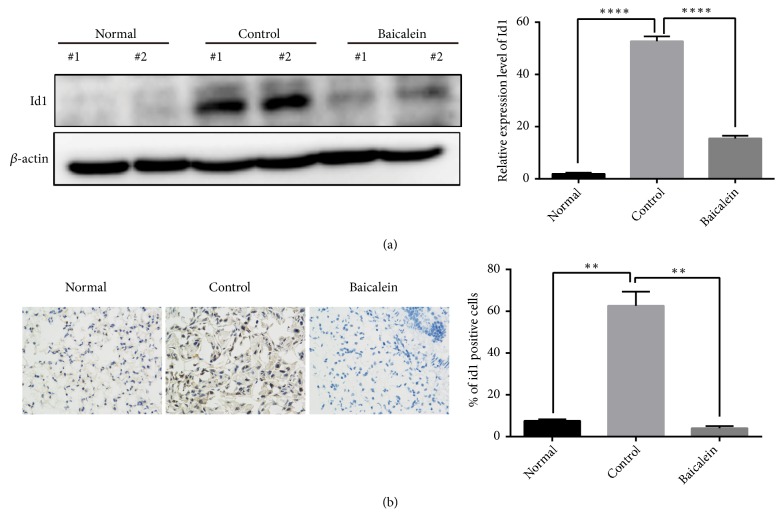
Baicalein inhibited Id1 expression of A549 orthotopic NSCLC xenografts. (a) Western blot analysis was performed to determine the Id1 protein. Two representative mice were presented. (b) Immunohistochemical analysis for Id1 of normal lung or xenograft tissues. The total number of Id1 positive cells (brown-stained nuclei, regardless of staining intensity, were counted as positive) in three random microscopic fields was counted by Image-Pro Plus 6.0. Data was shown as mean ± SEM (*∗∗P*< 0.01, *∗∗∗∗P*< 0.0001 compared with the control group).

To explore mechanism of the anti-tumor effect of baicalein, we detected Id1 expression in each group as Id1 is an essential tumor promoter which promotes the proliferation of tumor cells and facilitates tumor growth [17]. When compared with lungs of normal mice, lungs of tumor bearing mice expressed higher Id1 protein (*P*<0.0001) and baicalein significantly inhibited the Id1 expression level (*P*<0.0001) (Figure 3(a)). The immunohistochemistry also showed much more Id1 positive cells in control group (*P*<0.01); baicalein significantly reduced the number of Id1 positive cells (*P*<0.01) (Figure 3(b)).

### 3.3. Baicalein Abrogated Epithelial-Mesenchymal Transition (EMT) and Angiogenesis through Src/Id1 Signaling Pathway

**Figure 4:**
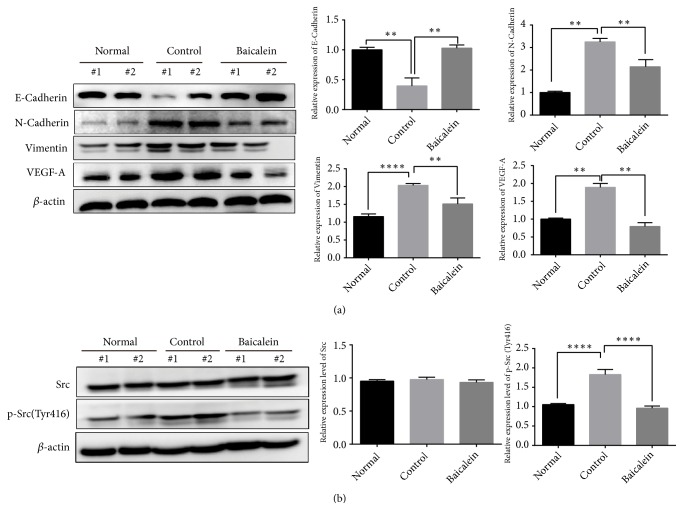
Baicalein suppressed the EMT procedure, VEGF-A, and phosphorylation of Src. Western blot was performed to analyze the (a) EMT related markers, VEGF-A, and (b) Src, p-Src (Tyr416). Two representative mice were presented. Data was shown as mean ± SEM, *∗P*<0.05; *∗∗P*<0.01; *∗∗∗∗ P*<0.0001.

Tissue microarrays containing 532 NSCLC patients' samples show that Id1 is significantly correlated with EMT-related proteins [10]. The loss of Id1 reduces the levels of EMT-related proteins [18] which indicate that EMT-related proteins may be the downstream of Id1 in NSCLC. Here, we obtained the inhibiting effect of baicalein to Id1 protein; thus we examined the expression of EMT-associated proteins (vimentin, E-cadherin, and N-cadherin). Results showed baicalein abrogated the increase of N-cadherin (*P*<0.01) and vimentin (*P*<0.01) and the decrease of E-cadherin in tumor bearing lung tissue (*P*<0.01) (Figure 4(a)). A study showed that Id1 has been implicated in VEGF-A regulation during tumor angiogenesis [19] and peritoneal expression of VEGF-A is regulated by TGF-*β*1 through the ID1 pathway in women with endometriosis [20]. In our research, we observed that VEGF-A was overexpressed in control group and baicalein significantly inhibited the expression of VEGF-A (*P*<0.01) (Figure 4(a)). To assess how baicalein inhibits the expression of Id1 protein, we analyzed the effect of baicalein to Src and its phosphorylation as studies show that Src regulates the expression of Id1 in human lung adenocarcinoma and pancreatic adenocarcinoma [7, 21]. Our results showed that baicalein significantly reversed high phosphorylation of Src in tumor bearing mice indicating that baicalein inhibits Id1 in an Src dependent manner (*P*<0.0001) (Figure 4(b)).

### 3.4. Regulation of Baicalein on Src/Id1 Signaling Pathway Was Verified In Vitro

**Figure 5:**
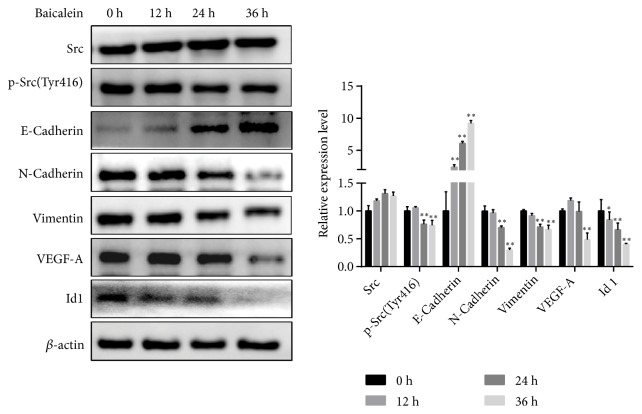
Baicalein regulated Src/Id1 signaling pathway. A549 cells were treated with baicalein (10 *μ*M) for 0 h, 12 h, 24 h, or 36 h and western blot was performed to determine expressions of p-Src (Tyr416), Id1, E-cadherin, N-cadherin, vimentin, and VEGF-A. Data was shown as mean ± SEM, *∗P*<0.05; *∗∗P*<0.01.

To verify the effect of baicalein to Src/Id1 signaling pathway, A549 cells were treated with baicalein (10 *μ*M) in different time points and expressions of p-Src (Tyr416), Id1, E-cadherin, N-cadherin, vimentin, and VEGF-A were determined by using western blot. As shown in Figure 5, baicalein inhibited Id1 expression time dependently. When A549 cells were treated with baicalein for 24 h or 36 h, the phosphorylation of Src and expressions of N-cadherin and vimentin were significantly suppressed (*P*<0.01). When treated for 36 h, VEGF-A was also reduced significantly. As for E-Cadherin, baicalein significantly upregulated its expression (*P*<0.0001).

## 4. Discussion

We established an orthotopic lung cancer model through a visible transthoracic injection with A549 cells and demonstrated that baicalein was effective in this model. We identified Id1 as the pivotal respondent to baicalein in its anti-tumor effect. The downstream molecules of Id1 (N-cadherin, vimentin, and VEGF-A) were further suppressed, and epithelial marker (E-cadherin) was increased. We also demonstrated that baicalein might function through inhibiting the phosphorylation of Src as it was the upstream of Id1. In light of our results, we propose baicalein as a promising adjuvant therapy phytochemical and Id1 and its mediators as candidates for targeted anti-tumor therapeutic strategies.

Nowadays, lung cancer is the most lethal cancer type in which NSCLC accounts for approximately 85% of all lung cancers [22, 23]. Except for chemotherapy drugs, a variety of other medicines, for instance, EGFR inhibitors, ALK inhibitors, angiogenesis inhibitors, and immune checkpoint inhibitors are available in the market [24]. However, despite the advances in therapies, the five-year survival of NSCLC remains low. This highlights the necessity for alternative treatments for unresectable NSCLC bearing patients. Baicalein is a main component of* Scutellaria baicalensis *which has been used to treat diseases for thousands of years in China. Properties of its effectiveness and low toxicity have gained admiration of patients. The anticancer potential of baicalein was observed in lung cancer, bladder cancer, breast cancer, ovarian cancer, etc. [25]. Nevertheless, most of these studies obtained the results through experiments* in vitro* or subcutaneous xenografts* in vivo *model. Our study firstly demonstrates its anticancer potential by using orthotopic lung cancer model which mimics the clinical situation preferably (Figure 2). This is of significant importance for preclinical drug research and provides more powerful evidence for baicalein of being a promising anticancer therapeutic drug.

Researchers have shown that baicalein may function through apoptosis induction, autophagy triggering, cell cycle arrest, or inhibition of 12-lipoxygenase (LOX) [12]. Our study identified Id1 as a critical respondent to baicalein for the first time. Id1 is a member of HLH family which functions as a differentiation inhibitor, and it is overexpressed in many cancers and facilitates the growth and metastasis of cancer [5]. In our study, tumor burden increased the expression of Id1 and baicalein significantly inhibited its expression time dependently (Figures 3 and 5), and thus proliferation and metastasis of tumor cells were suppressed. We also showed that mesenchymal markers (vimentin and N-cadherin) and angiogenesis marker (VEGFA) increased and epithelial markers (E-cadherin) significantly decreased in lungs of tumor bearing mice and baicalein regressed these effects* in vivo* and* in vitro*. This indicated that EMT procedure and angiogenesis of lung cancer were abrogated by baicalein. Studies have shown that Src is the upstream of Id1. We observed that baicalein could inhibit the p-Src (Tyr416) expression which indicated that its effect to Id1 might be through inhibiting Src phosphorylation (Figures 4 and 5).

In conclusion, our data demonstrated the antitumor effect of baicalein in orthotopic NSCLC model for the first time and clarified that Src/Id1 pathway was involved in the baicalein-induced inhibition of tumor growth, providing further insight into the therapeutic strategies.

## Data Availability

All data generated or analyzed during this study are included in this article.
